# Higher loss of livelihood and impoverishment in households affected by tuberculosis compared to non-tuberculosis affected households in Zimbabwe: A cross-sectional study

**DOI:** 10.1371/journal.pgph.0002745

**Published:** 2024-06-07

**Authors:** Collins Timire, Rein M. G. J. Houben, Debora Pedrazzoli, Rashida A. Ferrand, Claire J. Calderwood, Virginia Bond, Fredrick Mbiba, Katharina Kranzer

**Affiliations:** 1 Clinical Research Department, London School of Hygiene & Tropical Medicine, London, United Kingdom; 2 AIDS & TB Department, Ministry of Health and Child Care, Harare, Zimbabwe; 3 The Health Research Unit Zimbabwe, Biomedical Research & Training Institute, Harare, Zimbabwe; 4 Department of Infectious Disease Epidemiology, London School of Hygiene & Tropical Medicine, London, United Kingdom; 5 Department of Global Health and Development, Faculty of Public Health and Policy London School of Hygiene &Tropical Medicine, London, United Kingdom; 6 Social Sciences Unit, Zambart, Lusaka, Zambia; 7 Division of Infectious Diseases and Tropical Medicine, LMU University Hospital, LMU Munich, Germany; 8 German Center for Infection Research (DZIF), Munich, Germany; JIPMER PSM: Jawaharlal Institute of Post Graduate Medical Education and Research Department of Preventive and Social Medicine, INDIA

## Abstract

Tuberculosis (TB) disproportionally affects poor people, leading to income and non-income losses. Measures of socioeconomic impact of TB, e.g. impoverishment and patient costs are inadequate to capture non-income losses. We applied impoverishment and a multidimensional measure on TB and non-TB affected households in Zimbabwe. We conducted a cross-sectional study in 270 households: 90 non-TB; 90 drug-susceptible TB (DS-TB), 90 drug-resistant TB (DR-TB) during the COVID-19 pandemic (2020–2021). Household data included ownership of assets, number of household members, income and indicators on five capital assets: financial, human, social, natural and physical. Households with incomes per capita below US$1.90/day were considered impoverished. We used principal component analysis on five capital asset indicators to create a binary outcome variable indicating loss of livelihood. Log-binomial regression was used to determine associations between loss of livelihood and type of household. TB-affected households were more likely to report episodes of TB and household members requiring care than non-TB households. The proportions of impoverished households were 81% (non-TB), 88% (DS-TB) and 94% (DR-TB) by the time of interview. Overall, 56% (152/270) of households sold assets: 44% (40/90) non-TB, 58% (52/90) DS-TB and 67% (60/90) DR-TB. Children’s education was affected in 33% (55/168) of TB-affected compared to 14% (12/88) non-TB households. Overall, 133 (50%) households experienced loss of livelihood, with TB-affected households almost twice as likely to experience loss of livelihood; adjusted prevalence ratio (aPR = 1.78 [95%CI:1.09–2.89]). The effect of TB on livelihood was most pronounced in poorest households (aPR = 2.61, [95%CI:1.47–4.61]). TB-affected households experienced greater socioeconomic losses compared to non-TB households. Multisectoral social protection is crucial to mitigate impacts of TB and other shocks, especially targeting poorest households.

## Introduction

An estimated 10.6 million people fell ill with tuberculosis (TB) in 2022 and 1.1 million (10.3%) of them died [1]. Globally, around 400 000 people developed rifampicin resistant TB in 2022 (herein referred to as drug resistant TB [DR-TB]). The World Health Organisation (WHO) Africa, Western Pacific and South East-Asia regions account for 90% of global TB notifications [2]. TB is fuelled by HIV, with TB/HIV co-infection exceeding 50% in Zimbabwe, a country with an estimated incidence of TB of 204 per 100 000 population in 2022 [1, 2]. Treatment success for DR-TB averages around 60% globally and around 42% in Zimbabwe) [1, 3–5], compared to 90% for people with drug-susceptible TB (DS-TB) [1, 6]. TB disproportionally affects socioeconomically deprived people and leads to income and non-income losses [7]. While TB diagnostic tests and medicines are usually provided free-of-charge in public health institutions, hospitalisations, radiology services and blood tests are often not covered in low and middle income countries (LMICs) [8]. Households also experience social impacts of TB, (stigma, social exclusion, deterioration of relations with neighbours and landlords) and non-medical costs related to travel and food, in addition to income loss before, during and after TB treatment [9–14]. This, coinciding with reductions in household income, leads to severe socioeconomic burden [15].

The impact of TB on households is more pronounced in the context of DR-TB [7]. Historically, DR-TB treatment used to be 18–24 months long and people with DR-TB used to be hospitalised (e.g. for injectable medications), and often experienced severe disease partly due to treatment delays and complications. Treatment delays may result from delayed health seeking, barriers to accessing TB diagnostic tests and people being incorrectly started on DS-TB regimens before DR-TB is identified and people are switched to effective regimens [16]. Often people with extra-pulmonary TB incur huge medical costs related to radiology services (X-rays) and expensive diagnostic tests which are usually not available in public facilities [8]. This delays diagnosis and TB treatment. People with TB and their household members lose productive time during health seeking. Overall, the total TB costs fall within three categories: direct medical (consultations, X-rays); direct non-medical (transport, food) and indirect costs (income loss). The last two are the major drivers of catastrophic costs [9, 10]. TB affected households also experience stigma, social exclusion and worsening relations with family and neighbours. Extended family and neighbours are supportive structures which help TB affected households with interest free loans and assistance with household chores. Strained relations associated with TB disease reduce access to assistance from these support systems.

Estimating the socioeconomic impact of TB on households is challenging. Common measures of impact of TB are impoverishment and patient costs. The former determines the proportion of households that are pushed further into poverty by TB by comparing per capita income per day against a threshold, usually the international poverty line of United States Dollar (US$) 1.90 per person per day [17]. Patient costs surveys (PCS) collect data on total costs of TB (direct medical, direct non-medical and indirect costs) [18–20]. Global estimates of catastrophic costs, as measured through nationally representative PCS have revealed higher pooled prevalence of catastrophic costs in DR-TB (82%) than in DS-TB affected households (39%) [9, 21–24]. However, PCS are benchmarked against income, and this may overestimate the impact of TB among poor people, most likely to have unstable incomes.

TB affects all facets of human wellbeing, leading to income and non-income losses. The sustainable livelihood framework (SLF) [25], is a useful lens to inform multidimensional and holistic estimates of socioeconomic impacts of TB. The framework conceptualises that households live in a vulnerability context characterised by various shocks, and they utilise five available capital assets (human, financial, social, physical and natural capital) and various livelihood (coping) strategies to mitigate impacts of shocks [25]. Livelihood strategies are either accumulative or coping (survival) strategies in order to survive shocks [26–28]. Coping strategies may be harmful or non-harmful to livelihoods. Households may adopt short-term, non-harmful coping strategies e.g. spending savings, borrowing [29]. However, prolonged and/or sudden shocks may force households to expend resources rapidly and adopt harmful coping strategies e.g. taking loans at exploitative interest rates and selling assets [29–31]. Coping strategies determine the capital assets that are available in households, and whether households become resilient or vulnerable to shocks [31].

Quantitative measures based on the SLF have been used to study the impact of shocks on household livelihoods in the context of agroforestry and climate change [25, 32, 33]. A similar approach could be used to measure the impact of TB [34]. Zimbabwe, a LMIC has experienced economic challenges for a long time. The PCS in Zimbabwe revealed high proportions of catastrophic costs among DS-TB (79%) and DR-TB (90%) affected households [9], partly reflecting the harsh economic conditions in which people live and take their TB treatment. It is important to determine if the effects observed among TB affected households are attributable to TB. We apply impoverishment and our SLF-based measure [34], to assess socioeconomic impacts of TB on households overall, and stratified by DR-TB or DS-TB, compared to non-TB affected households in the same communities.

## Methods

### Study setting

This study was conducted in four provinces of Zimbabwe: Harare and Bulawayo (both predominantly urban), Masvingo (urban and rural) and Matabeleland South (predominantly rural) (S1 Fig). These provinces were purposively selected based on high DR-TB notifications. Zimbabwe, a southern African country, had a population of 15.1 million people in 2022 [35], and an estimated TB and DR-TB incidence of 204/100,000 population and 4.9/100,000 population in 2022 [1, 36, 37]. There are 10 provinces and 65 districts. Treatment success (completion and cure) was 83% for people with DS-TB and 54% among people with DR-TB [3, 38, 39]. The prevalence of TB/HIV co-infection was 50%. The prevalence of HIV in the general adult population is estimated at 12.9%, but is much higher (17.6%) in Matabeleland South province [40]. Zimbabwe has experienced socioeconomic challenges in the past two decades, with unemployment as high as 90% in 2015 [41]. In 2020, the Human Development Index was only 0.571, placing it 150^th^ out of 189 countries [42]. About 72% of Zimbabwean population live below the poverty line of US$1.90 per day [41].

This study was conducted during the COVID-19 pandemic (October 2020-March 2021) and as a result there were several COVID-19 waves and various degrees of national lockdowns during the study period. Zimbabwe recorded the first case of COVID-19 in March 2020, resulting in lockdowns where businesses were shut and health workers were reassigned to COVID-19 related work [43, 44]. The government’s social protection scheme, the Harmonised Social Cash Transfer, initially meant for food insecure households [45], was activated to cushion vulnerable households during lockdowns. The highest disbursement was US$25 per household per month. However this was converted to local currency equivalent at prevailing interbank rates which are often much lower than the black market rates on which most retail operates. Consequently, the US$25 disbursement was in fact worth very little: enough to buy five kilogrammes of maize flour which could feed a household of five people with their staple carbohydrate for at most a week.

#### Management of TB in Zimbabwe

TB treatment in Zimbabwe is decentralised to primary health facilities. TB services are integrated with HIV services. HIV services and TB molecular diagnostics, e.g. Xpert MTB/Rif assay (Cepheid, Sunnyvale, CA, USA), and treatment are provided free-of-charge. However, costs incurred prior to diagnosis, including clinic fees, hospitalisation costs, radiology investigations and laboratory tests are not covered. Radiology and many laboratory tests are mostly unavailable in public facilities and are usually accessed from private health providers, resulting in significant out-of-pocket costs. An all-oral 9-month DR-TB treatment regimen was introduced in 2021, replacing the longer 18–24 month injection-based regimen [46]. People on DR-TB treatment are eligible for non-contributory social protection in the form of conditional cash transfers (CCTs). Once registered, they receive US$25 per month till treatment completion, death or loss-to-follow up, whichever comes first. However, the cash transfer is subject to delays, unpredictable disbursements and has modest coverage [47].

#### Study design and population

In this cross-sectional study, adults (≥18 years) who were alive and on treatment for DR-TB and DS-TB at 35 selected health facilities (S1 Fig) during the study period were eligible for inclusion. Health facilities were selected based on DR-TB caseloads in 2018. The study team consecutively identified people who were alive and on DR-TB treatment from TB registers within sampled facilities. The registers were complete with respect to variables such as type of TB (DR-TB/DS-TB), HIV status, age and sex. Data, including age, sex, treatment regimen and mobile phone numbers were extracted from TB registers. For each person with DR-TB, an age (within 5 year age-bands) and sex-matched person with DS-TB was also identified from the same TB register. For example, a 25 year old DR-TB affected female was matched with a DS-TB affected female within the age range 20–30 years. Prospective participants were called by the study team who briefly described the purpose and procedures of the study and how the contact details for prospective participants had been obtained. Face-to-face meetings were arranged with those who expressed interest in the study. Those who were willing to participate were asked for written informed consent in local languages Shona or Ndebele.

Households from which community controls were selected were within 500 metres of the DR-TB affected households. Hereafter, these households are referred to as ‘non-TB households’. To protect confidentiality, five households neighbouring DR-TB affected households on the same street in either direction were not approached for participation. Community controls were also matched for age and sex with those affected by DR-TB and DS-TB in the ratio 1:1:1.

#### Data collection

Data were collected using interviewer administered paper-based individual and household questionnaires. If the person with TB was the head of household, a household questionnaire was also administered, otherwise consent was sought from the head of household to administer a household questionnaire. Individual questionnaires captured socioeconomic details, experiences of stigma, duration from onset of TB symptoms to diagnosis of TB, past medical history, money spent on travel and medical expenses, income at time of interview, receipt of any social protection (only for people with DR-TB), type of social support required (only for people with DS-TB and DR-TB), any relocation and physical fitness. Stigma was measured using the scale adapted by Marangu et al. [48]. The scale has 13 items capturing internalised stigma (4 items), perceived stigma (4 items) and general stigma (5 items). Each item was measured on a Likert scale ranging from 0–4: 0 indicating “Never”; 1 “Rarely”; 2 “Occasionally”; 3 “Regularly” and 4 indicating “Always”. Household questionnaires included questions on type of household, number of household members, household asset ownership, current income, size of household, dissavings (sale of assets, spending savings and borrowings), failure to repay loans, pledging crops or cattle, whether children were transferred to cheaper schools, whether children were withdrawn from schools among the households that had school going children, changes in relations with family/neighbours, whether a household member required caregiving, changes of head of household and deaths in the household. The variables in the household questionnaire were informed by the SLF [25], variables in patient cost surveys [49], and indicators adapted from a study investigating livelihood in the context of HIV in Zimbabwe [50]. The SLF indicators are presented in S1 Table. All interviews were held in private locations suggested by participants.

### Data analysis

Data were entered in EpiData v3.1 (EpiData Association, Odense, Denmark) and were exported to Stata version 13 (StataCorp, College Station, TX, USA) for cleaning and analysis. The exposure of interest was type of household: non-TB affected or TB affected household. TB affected households were further categorised into DS-TB and DR-TB households. Categorical variables were summarised using frequencies and proportions. Differences in proportions were compared using the chi-square test. Continuous variables were summarised using medians and interquartile ranges (IQRs) and differences were compared using the Mann-Whitney-U test. Some variables were derived during analysis. These include effect on education of children (which was a composite of withdrawal of children from school or transferring children to cheaper schools or both);loss of household income (binary variable (yes/no) was determined when income at time of study was lower than income 12 months before the interview); dissavings (sale of assets, spending savings, taking loans); and changes in social relations comparing 12 months before and at the time of the interview based on self-reports by participants using Likert scales ranging from 1–10. A reduction in score was reflective of deteriorating social relations. To calculate impoverishment, we divided monthly household income by 30.5 and by the number of household members to determine income per person per day and classified households as impoverished when income per capita was below the poverty line of US$1.90 per day [51]. Monetary values reported in South African Rand (commonly used in Masvingo and Matabeleland South provinces) were converted to US$ for calculations using the Oanda currency converter (http://www.oanda.com). Total stigma was calculated by averaging the scores for the 13 scale items and individuals were considered to have experienced stigma when the average score was ≥2. We calculated mean scores for each of the five capital indicators and presented results as spider diagrams. We used principal component analysis (PCA) to categorise households into tertiles (poorest, poor and not-so-poor) based on household asset ownership and to reduce data on the five capital assets and coping strategies into a dichotomous outcome variable indicating loss of livelihood as previously described [34]. Log binomial regression was used to test associations between loss of livelihood and type of household. We adjusted the analysis for a household member requiring a carer during the period 12 month prior to the interview in a multivariable Poisson regression, excluding matching variables (age, sex and province), and presented results as prevalence ratios (PRs) and adjusted PRs.

### Ethics

Ethical approval was obtained from the London School of Hygiene & Tropical Medicine Research Ethics Committee (22579), the Biomedical Research and Training Institute Institutional Review Board (AP160/2020) and the Medical Research Council Zimbabwe (MRCZ/A/2645). Permission to access TB registers was obtained from the Secretary for Health in the Ministry of Health ad Child Care Zimbabwe. All participants gave written informed consent to take part in the study.

## Results

We approached 285 people, of whom 270 (95%) people (and all corresponding heads of households) consented to take part in the study. Non-TB affected participants were less likely to be living with HIV (30% vs 66%), to report hospitalisations (2% vs 27%) and to have relocated 12 months prior the study (9% vs 34%) compared to TB-affected participants. People on DR-TB treatment incurred 2.7 times higher TB-related costs than people on DS-TB treatment (p<0.001). Of the 62/90 people with DR-TB who registered for CCTs, 40 (65%) reported receiving any cash disbursement. TB stigma was experienced by 22 (24%) of people with DR-TB compared to 12 (13%) people with DS-TB (p = 0.06) (Table 1).

**Table 1:** Characteristics of participants (individual-level questionnaire) who were enrolled in the study.

Characteristic		Non-TB N = 90	DS-TB N = 90	DR-TB N = 90	p-value
		n (%)	n (%)	n (%)	
Province	Harare	22 (24%)	22 (24%)	22 (24%)	1.00
	Bulawayo	24 (27%)	24 (27%)	24 (27%)	
	Matabeleland South	24 (27%)	24 (27%)	24 (27%)	
	Masvingo	20 (22%)	20 (22%)	20 (22%)	
Men		50 (56%)	50 (56%)	50 (56%)	1.00
Age category	≤24	7 (8%)	12 (13%)	12 (13%)	0.64
	25–34	27 (30%)	30 (33%)	24 (27%)	
	35–44	33 (37%)	33 (37%)	36 (40%)	
	45–54	14 (16%)	12 (13%)	10 (12%)	
	55+	9 (10%)	3 (3%)	7 (8%)	
Education of person	Primary¥	8 (9%)	17 (19%)	25 (28%)	0.03
	Secondary⁑	72 (80%)	66 (73%)	59 (65%)	
	Tertiary*	10 (11%)	7 (8%)	6 (7%)	
HIV^1^ positive		27 (30%)	58 (64%)	60 (67%)	<0.001
Previous history of TB		14 (16%)	10 (11%)	26 (29%)	0.01
Phase of TB treatment	Intensive	N/A	51 (57%)	17 (19%)	<0.001
	Continuation	N/A	39 (43%)	73 (81%)	
Experienced TB stigma	Yes	N/A	12 (13%)	22 (24%)	0.06
Interval from symptoms to diagnosis (weeks), [median (IQR)]		N/A	9 (6–22)	13 (5–24)	0.29
Hospitalization in the past 12 months		2 (2%)	18 (20%)	30 (41%)	<0.001
Loss of income in the past 12 months		46 (51%)	74 (82%)	79 (88%)	<0.001
Changed residency in the past 12 months		8 (9%)	29 (32%)	33 (37%)	<0.001
TB related costs (US$) [Median (IQR)]		N/A	150 (100–275)	400 (244–728)	<0.001
Registered for cash transfers		N/A	N/A	62 (69%)	
Type of social support preferred:					
Food and cash		N/A	39 (43%)	52 (58%)	0.33
Cash/cash vouchers		N/A	28 (31%)	20 (22%)	
Food/food vouchers		N/A	21 (23%)	17 (19%)	
Food and counselling		N/A	1 (1%)	1 (1%)	

SES = Socioeconomic status; HH = Household; DS-TB = drug susceptible tuberculosis; DR-TB = drug resistant tuberculosis; IQR = interquartile range; US$ = United States Dollar,

^1^ 1 = unknown;

^¥^ Primary education = First 7 years of formal education after kindergarten level;

^⁑^ Secondary education = second stage of formal education. It encompasses year 8–13 of formal education;

* Tertiary education = university or polytechnic college education.

Across the three strata, households were similar with respect to sex of head of household, socioeconomic status, education of head of household and the percentage having experienced a death in the household over the past 12 months. Of the 52 households that experienced deaths, a COVID-19 related death was reported in one household. A higher proportion of TB-affected households had experienced TB before (44% vs 31%) and reported that a household member had needed to be taken care of 12 months prior to the interview (72% vs 22%), compared to non-TB households (Table 2).

**Table 2:** Characteristics of study households that were enrolled in the study.

Characteristic		Non-TB N = 90	DS-TB N = 90	DR-TB N = 90	p-value
Setting	Urban	50 (56%)	60 (67%)	55 (61%)	0.31
	Rural	40 (44%)	30 (33%)	35 (39%)	
Socioeconomic status (n = 258)	Poorest	31 (38%)	28 (32%)	34 (39%)	0.46
	Poor	22 (27%)	27 (30%)	30 (35%)	
	Not so poor	29 (35%)	34 (38%)	23 (26%)	
*Head of household*					
Men		58 (64%)	54 (60%)	62 (69%)	0.46
Age	≤24	3(3%)	4(4%)	5 (6%)	0.76
	25–34	31(35%)	21(24%)	19 (21%)	
	35–44	30 (33%)	37 (42%)	40 (44%)	
	45–54	16 (18%)	20 (22%)	17 (19%)	
	55+	10 (11%)	7 (8%)	9 (10%)	
Education	≤Primary	11 (12%)	18 (20%)	23 (25%)	0.29
	Secondary	69 (77%)	64 (71%)	61 (68%)	
	Tertiary	10 (11%)	8 (9%)	6 (7%)	
*Health of members of the household*					
Previous history of TB in household		28 (31%)	35 (39%)	45 (50%)	0.03
Death of a member*		15 (16%)	17 (19%)	20 (22%)	0.64
Member requiring care*		20 (22%)	60 (67%)	70 (78%)	0.001
*Coping strategies*					
Withdrew/transferred children		12 (14%)⁑	27 (34%)§	28 (32%)¥	0.01
• Withdrew children from schools		3 (3%)	18 (21%)	23 (26%)	
• Moved children to cheaper schools		12 (14%)	16 (18%)	9 (10%)	
Sold assets		40 (44%)	52 (58%)	60 (67%)	0.01
Spent savings		49 (54%)	68 (76%)	64 (71%)	0.01
Borrowed		52 (58%)	65 (72%)	69 (77%)	0.02
Experienced income loss		48 (53%)	74 (82%)	81 (90%)	0.001
Failed to repay loans		16 (18%)	37(41%)	49 (54%)	0.001
Perceived impact of shocks on HH during the past 12 months	Severe	32 (36%)	63 (70%)	83 (92%)	<0.001
	Moderate	28 (31%)	21 (23%)	6 (7%)	
	Little/no impact	30 (33%)	6 (6%)	1 (1%)	
Living under poverty line (poverty line US$ 1.90)		73 (81%)	78 (88%)	85 (94%)	0.02
Living under poverty line (poverty line US$ 2.15)		78 (87%)	79 (89%)	85 (94%)	0.20
Experienced worsening social relations		23 (26%)	43 (48%)	45 (50%)	<0.001

DS-TB = Drug susceptible tuberculosis; DR-TB = Drug resistant TB;

* = death in the past 12 months. Of the 52 people who died, the distribution of deaths was as follows: Chronic illness = 25 (48%); Short illness = 10 (19%); TB = 7 (13%); Road traffic accident = 6 (12%); Other = 3 (6%) and COVID-19 = 1 (2%); US$ = United States Dollar; HH = Household;

^⁑^ denominator was 88 non-TB households;

^§^ = denominator = 80 DS-TB households;

^¥^ = denominator = 88 DR-TB households.

The number (proportion) of impoverished households was 73/90 (81%) among non-TB, 78/90 (88%) DS-TB and 85/90 (94%) DR-TB households (p = 0.02)TB-affected households experienced higher dissavings (borrowing, selling assets, spending savings) as compared to community households (Table 2). Overall, 56% (152/270) of households sold assets: 44% (40/90), 58% (52/90), 67% (60/90) of non-TB, DS-TB and DR-TB households, respectively (p = 0.01). Median TB-related costs were higher among DR-TB households compared to DS-TB and non-TB households (DR-TB: US$400 [IQR:244–728] vs DS-TB: US$150 [IQR:100–275], p<0.001; Table 2) A third of TB-affected households (n = 55, 33%) reported that the education of children was negatively affected compared to one in seven (n = 12, 14%) of non-TB households. Heads of households in 83 (92%) DR-TB and 63 (70%) DS-TB households reported their livelihoods were severely affected in the past 12 months compared to 32 (36%) heads of non-TB households.

**Fig 1:**
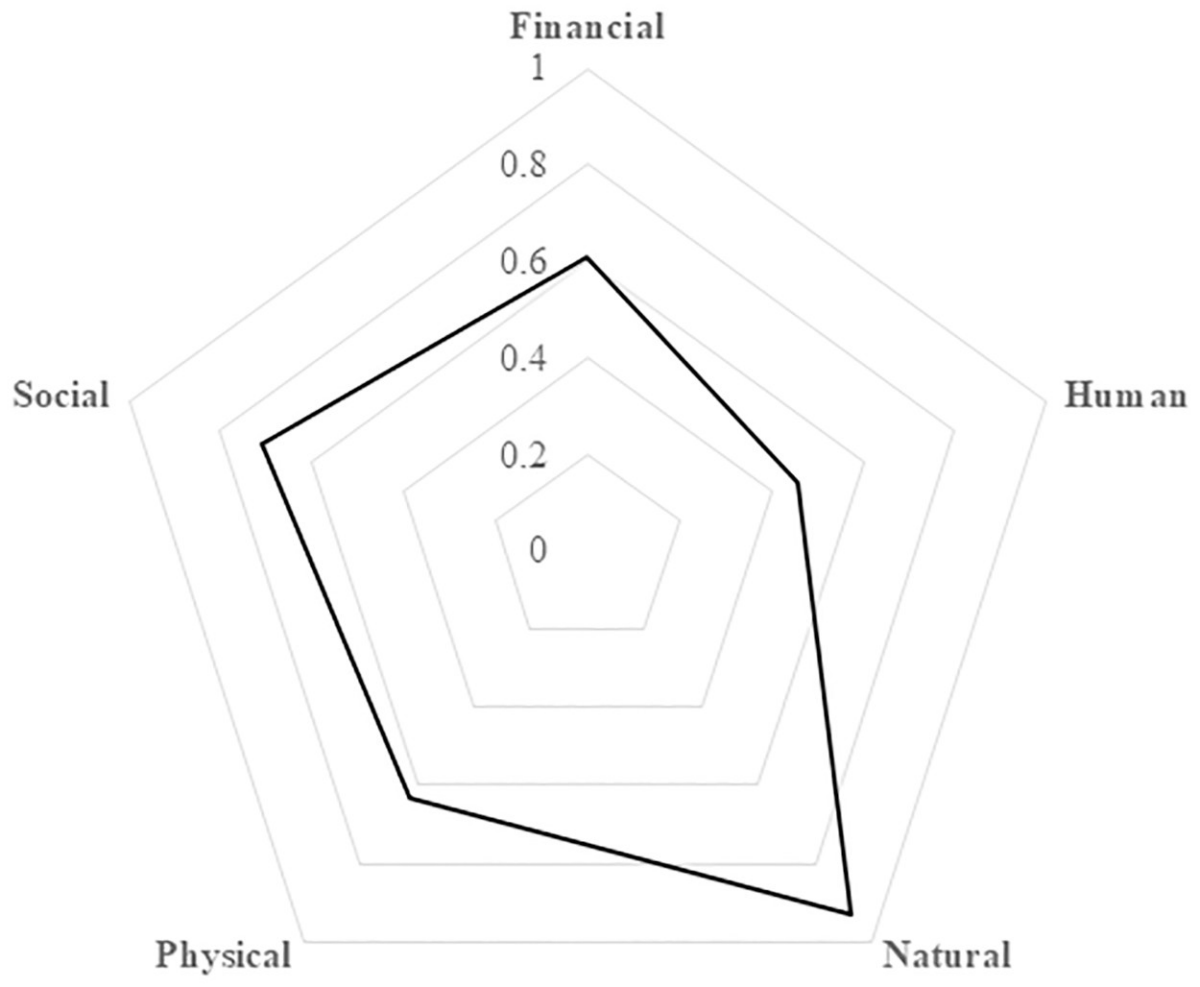
Spider-plot showing how shocks such as TB and COVID-19 affected the five capital assets in all households. Fully resilient households have a score of 1 in all the five capital assets and when the whole area of the pentagon is covered. Vulnerable households have low scores in most or all the five capital assets. Accordingly the area of the pentagon covered will be small.

**Fig 2:**
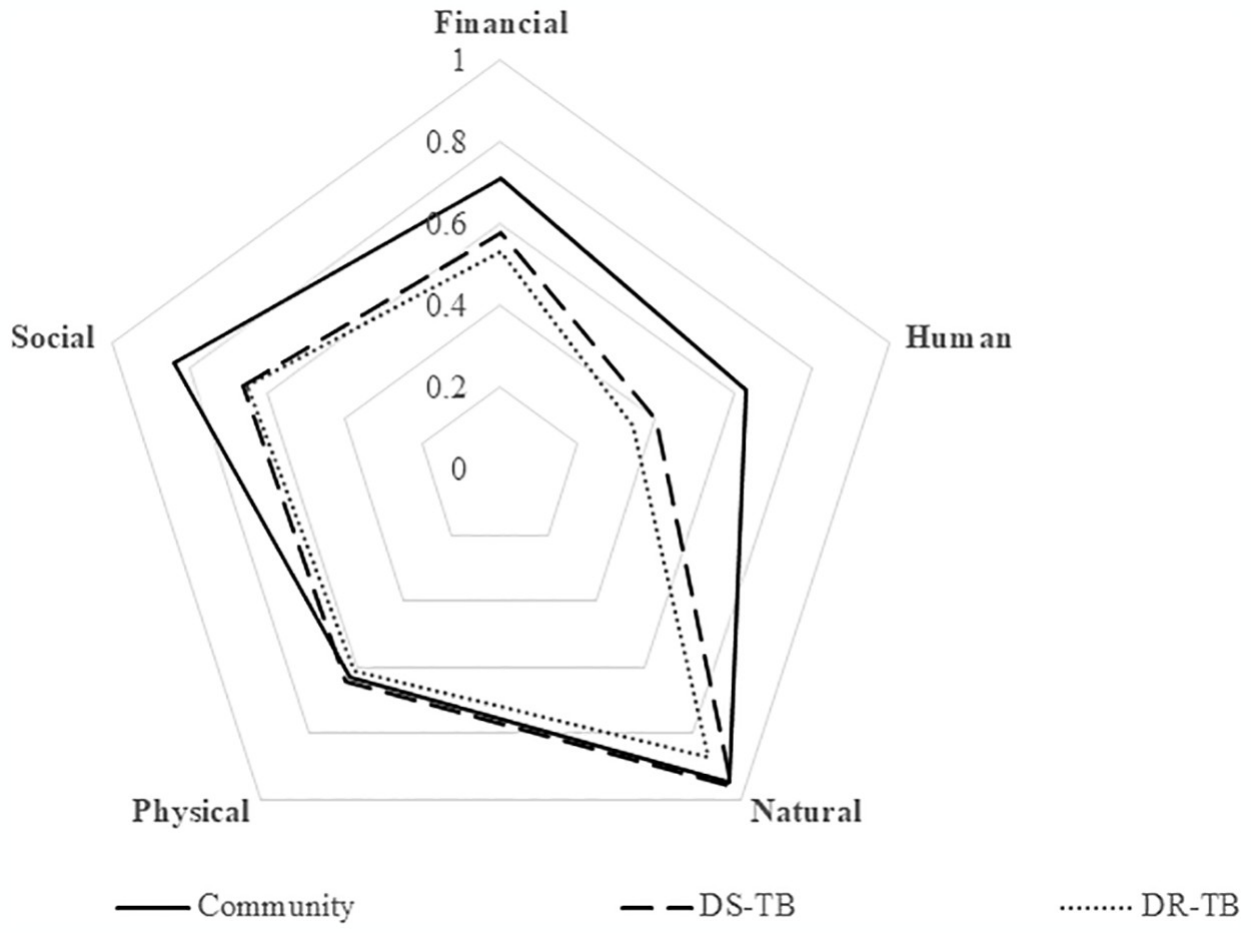
Spider-plot showing how shocks such as TB and COVID-19 affected the five capital assets, stratified by type of household. DS-TB = drug susceptible TB; DR-TB = Drug resistant TB. Fully resilient households have a score of 1 in all the five capital assets and when the whole area of the pentagon is covered. Vulnerable households have low scores in most or all the five capital assets. Accordingly the area of the pentagon covered will be small. Non-TB affected households have more capital assets (cover more area of the pentagon) than TB affected households (DR-TB and DS-TB).

Huge impacts on financial, human and social capitals were experienced in TB-affected compared to non-TB households (Figs 1 and 2). Overall, 133 (50%) [95% confidence interval (CI): 44%-56%]) of households experienced loss of livelihood. Loss of livelihood was higher in DR-TB (62%) and DS-TB (60%) affected households compared to non-TB households (27%). TB affected households were almost two times more likely to experience loss of livelihood as compared to non-TB households, after adjusting for history of household member requiring a carer during the 12 month prior to the interview (adjusted prevalence ratio (aPR = 1.78 [95%CI:1.09–2.89]). There were no differences in loss of livelihood comparing DR-TB and DS-TB households (Table 3). The proportion of households experiencing loss of livelihood was 60% in the poorest households compared to 33% in the not-so-poor households (Table 3). In the stratified analysis, the effect of TB on loss of livelihood was worst in poorest households (PR = 2.61 [95%CI:1.47–4.61]), Table 3) compared to the not-so-poor households.

**Table 3:** Loss of livelihood in DS-TB, DR-TB affected households and non-TB households.

			Experienced loss of livelihood		
Characteristic		Total	Yes (%)§	PR 95% CI	aPR⁑ 95% CI
Total		268	133 (50)		
Type of household	DR-TB	90	56 (62)	2.31 (1.58–3.37)	1.78 (1.05–3.01)
	DS-TB	89	53 (60)	2.21 (1.51–3.24)	1.78 (1.06–2.98)
	Non-TB	89	24 (27)	Reference	Reference
Household member requiring a carer*	Yes	150	96 (64)	2.04 (1.52–2.74)	1.61 (1.06–2.45)
	No	118	37 (31)	Reference	Reference
Socioeconomic status	Poorest	93	56 (60)	1.81 (1.28–2.55)	
	Poor	89	45 (51)	1.71 (1.19–2.44)	
	Not so poor	84	28 (33)	Reference	
Effect modification**					
Crude PR comparing TB with Non-TB households				2.26 (1.57–3.24)	
PR stratified by socioeconomic status	Poorest			2.61 (1.48–4.61)	
	Poor			2.10 (1.11–3.97)	
	Not so poor			1.50 (0.72–3.10)	

* = in the last 12 months

^§^ = Row percentages;

PR = prevalence ratio; aPR = adjusted prevalence ratio; CI = confidence interval; DS-TB = drug susceptible tuberculosis; DR-TB = drug resistant tuberculosis;

^⁑^ = adjusted for household member requiring a carer;

** Comparing TB to non-TB households.

## Discussion

We used impoverishment and a multidimensional measure informed by the SLF to investigate socioeconomic impacts of TB on households. We found that TB-affected households experience greater impoverishment and loss of livelihood than non-TB households. There was no difference in loss of livelihood between DR-TB and DS-TB affected households. Socioeconomic status was an effect modifier, and the effect of TB on loss of livelihood was worst in poorest households.

These results are in line with studies conducted in Ghana and the Philippines showing that the proportion of impoverished households is higher among TB-affected compared to non-TB households [10, 52]. Of note in our study the proportion impoverished (81%) was extremely high even among non-TB households. This may partly be explained by the dire socioeconomic situation in Zimbabwe overall and in these communities. For the past two decades, Zimbabwe has experienced a sustained economic decline resulting in hyperinflation and out migration of skilled workers [53]. Droughts, floods and the COVID-19 pandemic have enhanced the existing economic challenges [54]. In 2019, an estimated 38.9% of the Zimbabwean population lived below US$2.15 per person per day [55]. Importantly, we used the pre-2017 poverty line (US$1.90). Using a cut-point of US$2.15 per person per day, 87%, 89% and 94% of non-TB, DS-TB and DR-TB households would have been categorised as impoverished.

Most studies on socioeconomic impact of TB rely on measuring income or costs [10, 11, 17]. Our study shows that financial capital (income, spending of savings to cover TB associated costs) is not the only livelihood capital that is affected by TB. The impact of TB was found to be more pronounced on human, social and financial capitals while physical and natural capital assets remained relatively stable across all households. Natural capital is setting-specific and likely more relevant in rural areas, whilst disposal of physical capital or dilapidation of physical capital as a result of reduced maintenance may be a strategy of last resort and only employed when shocks become chronic.

Household coping strategies evolve from short term e.g. dissavings (spending savings, borrowing) to long term coping strategies (withdrawal of children from school, sale of assets). As a result, cross sectional studies, especially those in which data are collected during the intensive phase of treatment (i.e. shortly after diagnosis), may not capture long-term coping strategies. The exception may be in extremely vulnerable households, which are likely to exhaust short-term coping strategies quickly and proceed to selling assets and/or abandon treatment [30, 56]. Long-term coping strategies are the most harmful to livelihoods, with greater, long-lasting impacts. This may force households into financial catastrophes and inter-generational poverty [57, 58]. Socioeconomic impacts of TB persist even after completing treatment as households continue to borrow and pledge their assets [13, 59, 60]. Livelihood is therefore dynamic since households experience shocks continuously and are actively utilising various coping strategies in their quest to maintain well-being [25]. For this reason, longitudinal studies including the post-TB treatment period are recommended as they are likely to provide more accurate estimates of the impact of TB on households [61].

Until recently, DS-TB and DR-TB treatment were different with regards to duration and toxicities. However, with roll out of shorter and all oral DR-TB regimens [62, 63], the differences are less pronounced. This may explain the lack of difference in loss of livelihood between DS-TB and DR-TB households. It is also possible that the impact of DR-TB was mitigated by the cash transfers that were provided to people with DR-TB. Around 69% were registered for cash transfers during the course of treatment. However, the proportion which received CCTs could not be established as there are delays in CCT disbursements [47]. Data from our study show that people with TB prefer cash and food to either cash alone or food alone. Nevertheless, DR-TB affected households were more likely to report severe impacts of TB and other shocks on their livelihoods compared to DS-TB. This is despite no differences in the proportion experiencing loss of livelihood.

The strengths of our study include recruitment of participants across four provinces in Zimbabwe, including both urban and rural sites, and investigating socioeconomic impact of TB using a multidimensional measure which is not benchmarked against income. In parallel, we used a more conventional measure (i.e. impoverishment) allowing direct comparisons between these two. The study was undertaken during the COVID-19 pandemic, a time of extreme socioeconomic vulnerability which may explain the high level or impoverishment in general and provides insight into the interaction between TB and other generalised socioeconomic shocks. This has important implications for pandemic preparedness policies [64], as it highlights the long-term impacts of pandemic responses on socioeconomic vulnerability and importance of providing social support to households during times of crisis. None of the households received the harmonised social cash transfers, a form of social protection aimed at cushioning them against COVID-19. Since TB is an extra shock to those experienced in the community, more support should be directed to TB affected households.

Our cross-sectional design made it impossible to capture changes in livelihood across all phases of TB treatment. Hence, there is potential underestimation of loss of livelihood, especially among people who were interviewed during early stages of TB treatment. We relied on self-reports of coping strategies and income. While coping strategies are unlikely to be influenced by recall bias, income often is. Income is difficult to reliably estimate especially in contexts characterised by informal/seasonal jobs [65]. We potentially underestimated loss of livelihood by enrolling people who were alive and on treatment, excluding those who died of TB or were lost to follow-up prior to the study. TB-related deaths result in huge costs of up to 15 times the monthly household income [29]. People who died or were lost to follow-up are likely to have experienced greater loss of livelihood than those who were alive and on treatment. Further, we cannot rule out possible overmatching of community controls because people who live in the same area as TB-affected households often have a similar socioeconomic background. The fact that 83% of non-TB households were living under the poverty line suggest that overmatching by socioeconomic status is likely. We purposively selected four out of the 10 provinces in Zimbabwe. Hence, our results may not be generalised to the whole of Zimbabwe.

Lastly, our study was conducted during the COVID-19 pandemic. Hence, both impoverishment and loss of livelihood due to TB are likely to be overestimated by this concurrent shock. COVID-19 increased economic burden of patients and communities mainly related to income losses and hospitalisations [44, 66–68].

Despite these limitations, our study has implications for policy and practice. Firstly, in a time of huge socioeconomic vulnerability (i.e. COVID-19), TB was associated with worse socioeconomic effects, especially in the poorest households. Hence, poorest households should be prioritised for multisectoral social protection to reduce the incidence and impacts of TB. People in the poorest households are more likely to experience food insecurity and malnutrition. Malnutrition increases risk of i) infection by *Mycobacterium tuberculosis*, ii) severe TB and iii) mortality [69]. Recent studies have shown that social protection in the form of a nutritional intervention reduces TB mortality and averts 40–50% of TB diseases [70, 71]. Secondly, this study highlights the importance of multidimensional measures to adequately capture income and non-income impacts of TB, including the effect of TB on schooling and ownership of assets, for programmatic action [16, 72].

## Conclusion

TB affected households experienced greater loss of livelihood than households currently not affected by TB. The effect of TB was most profound among the poorest households. Multisectoral approaches to support poorest households are crucial to mitigate the impact of TB.

## Supporting information

S1 Table Indicators contributing to the livelihood variable. † = A derived variable obtained from either moving children to cheaper schools and/or withdrawing children from school; ‡ = Coping strategies.
(DOCX)

S1 Fig Study sites. The map was created in R and the source files (shape file base maps) are from here Zimbabwe—Subnational Administrative Boundaries—Humanitarian Data Exchange (humdata.org).
(TIF)

S1 Data (CSV)

S1 Text Codebook. (TXT)
