# Supplementary material for: Higher loss of livelihood and impoverishment in households affected by tuberculosis compared to non-tuberculosis affected households in Zimbabwe: A cross-sectional study
Source: PLOS Glob Public Health. 2024 Jun 7;4(6):e0002745. doi: 10.1371/journal.pgph.0002745 (PMC11161058; doi:10.1371/journal.pgph.0002745)
Supplement: S1 Table — † = A derived variable obtained from either moving children to cheaper schools and/or withdrawing children from school; ‡ = Coping strategies. (DOCX) [file pgph.0002745.s001.docx]

| Livelihood capital | Indicators |
| --- | --- |
| Financial capital | - Sale of assets.‡ - Spending savings.‡ - Failure to repay loans. - Pledging future crops/cattle/livestock‡ - Borrowing at exorbitant interest rates‡ - Reduction in household income. |
| Physical capital | - Sale of productive assets.‡ - Failure to replace productive assets. |
| Social capital | - Changes in relationship with neighbours for the worse. - Changes in relationship with family members for the worse. - Received support from neighbours/family. |
| Natural capital | - Reduction in land that is farmed (idle land/leasing). - Sale of land/cattle to finance TB. |
| Human capital | - Change in head of household (death/abandonment by spouse/family). - Physical debility resulting in loss of labour for household. - Insufficient food. - Education of children affected**†** |
