# Supplementary figures and images for: Higher loss of livelihood and impoverishment in households affected by tuberculosis compared to non-tuberculosis affected households in Zimbabwe: A cross-sectional study
Source: PLOS Glob Public Health. 2024 Jun 7;4(6):e0002745. doi: 10.1371/journal.pgph.0002745 (PMC11161058; doi:10.1371/journal.pgph.0002745)

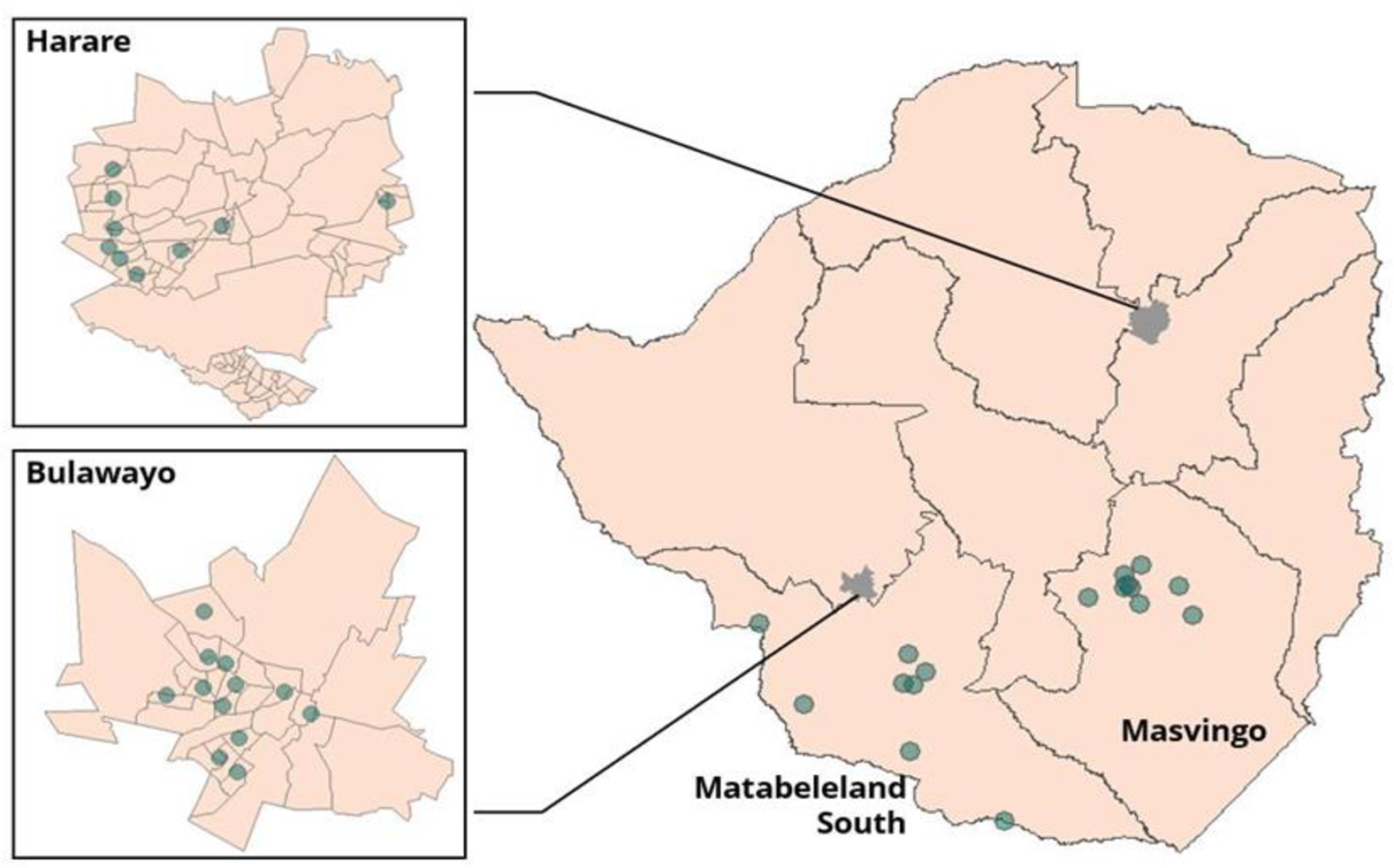

Supplement: S1 Fig — The map was created in R and the source files (shape file base maps) are from here Zimbabwe—Subnational Administrative Boundaries—Humanitarian Data Exchange (humdata.org). (TIF) [file pgph.0002745.s002.tif]
